# Charles Bonnet Syndrome as Another Cause of Visual Hallucinations

**DOI:** 10.7759/cureus.12922

**Published:** 2021-01-26

**Authors:** MaryKate Voit, Brian Jerusik, Justin Chu

**Affiliations:** 1 Emergency Medicine, Inspira Medical Center, Vineland, USA

**Keywords:** visual hallucination, cerebral vascular accident, occipital lobe infarct, hallucinations, stroke, case report

## Abstract

Charles Bonnet syndrome (CBS) presents as gradual vision loss and associated visual hallucinations in a patient who is otherwise neurologically and psychiatrically intact. This syndrome presents primarily as ophthalmologic disease, however, may be secondary to an ischemic stroke or tumor in the occipital lobe. Patients present with a complaint of vivid visual hallucinations ranging from spots and geometric shapes to seeing people, distorted figures and landscapes.First noted in the 1760s, CBS did not reach the western scientific community until the early 1980s.

Our patient reported seeing her dog and deceased mother only when looking left. She was having an experience of phantom images in addition to a visual field impairment but was otherwise of sound mind with no gross neurological deficits. Computer topography of the brain revealed a subacute infarct in the right posterior occipital lobe. The patient was ultimately diagnosed with CBS following magnetic resonance imaging and ophthalmology consultation at tertiary center.

Diagnosis may be delayed by lack of symptom reporting as patients do not want to carry a stigma as ‘crazy.’ Further, physician awareness of this etiology is low and a better understanding of the disease will prevent missed diagnosis as well as lack of appropriate consultation and follow-up. Treatment includes close outpatient ophthalmology care, maximizing existing vision and lifestyle changes including adjustments in lighting, decreasing stress and increasing socialization. Trials of prescription treatment (i.e., antipsychotics, serotonin reuptake inhibitors, antiepileptics) have shown only anecdotal evidence at efficacy. CBS is an uncommon presentation of cerebral vascular disease that warrants the attention of emergency department physicians.

## Introduction

Charles Bonnet syndrome (CBS) is defined as the occurrence of phantom vision in people living with some form of ophthalmologic disease who are otherwise cognitively and physiologically healthy. The condition was first noted by Charles Bonnet in 1760 in Geneva, Switzerland. Bonnet was a natural scientist, the youngest member of the Parisian Academy of Sciences and elected into the Royal Society of London. Unfortunately, he slowly began to lose his vision in his 20s making continued microscope use difficult prompting him to spend the second half of his life studying psychology, philosophy and metaphysics. Bonnet's published works reference his sane elderly grandfather and the 'visions' he would describe. Bonnet ultimately suffered similar hallucinations and continued visual loss late in his own life. The diagnosis was coined by neurologist, George de Morsier in the 1930s to describe visual hallucinations in the elderly whose insight remains intact [1]. Common ophthalmologic diseases associated with Charles Bonnet syndrome are macular degeneration and glaucoma as they alter stimuli within the visual cortex [2]. The core features of CBS involve vision impairment, existence of phantom images or visions, full or near full insight into the unreal nature of what one sees, no discernible cognitive memory deficits and visual images that do not extend to other sense modalities [1]. In less common cases, the syndrome can develop as a consequence of other clinical conditions such as brain surgery, multiple sclerosis, a tumor or in this case, an ischemic stroke. The following case report will discuss the presentation and diagnosis of CBS in the emergency department.

## Case presentation

A 68-year-old female with a history of hypertension and diabetes presented to the emergency department (ED) with complaints of left visual field hallucinations. The symptoms had begun four days ago. She reported, “if I look to my left, I see my dog, but I know he is not there.” The patient also reported seeing her deceased mother only in her left visual field. She stated that if she looked to her right, she no longer saw the hallucinations. She denied any motor or tactile sensory deficits. Patient’s neurologic baseline otherwise was awake, alert and oriented with no focal motor or sensory deficits. The patient did report right upper lid droop since her elective bilateral blepharoplasty one month ago.

Exam revealed an afebrile patient with a normal heart rate at 66 bpm, elevated blood pressure of 197/99 mmHg and oxygen saturation of 97% on room air. Visual acuity showed OD: 20/40, OS: 20/60. The patient was an overall well appearing female, awake and alert in no acute distress. Her exam was unremarkable other than pertinent findings in the eye and neurologic exam. Eye exam showed bilateral pupils equal, round and reactive to light. Extraocular movement was intact however mild right-sided ptosis was noted, reportedly chronic per patient secondary to recent blepharoplasty. Patient’s neurologic exam revealed an awake, alert and oriented patient to person, place, time and purpose with structured, linear mentation. All cranial nerves were intact and no dysmetria was noted. No sensory deficits or motor deficits were found on examination in all four extremities. The patient was noted to have visual field loss in the left peripheral field.

**Figure 1:**
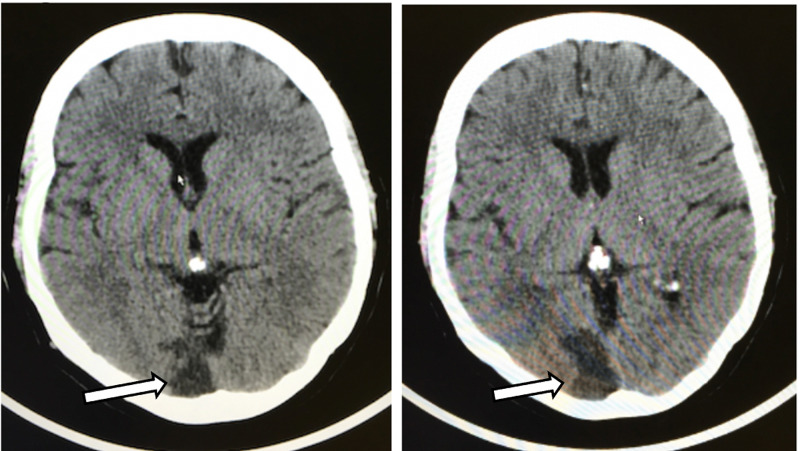
Computer Tomography of Brain Computed tomography of brain without contrast demonstrating right posterior infarction (white arrows).

Evaluation in the ED included a complete blood count, coagulation studies, troponin and alcohol level which were within normal limits. Both glucose and potassium were minimally elevated. Patient’s chest X-ray demonstrated no active disease and electrocardiogram showed a normal sinus rhythm with no arrhythmias or signs of ischemia. Evaluation also included neuroimaging (Figure 1).

Computed tomography (CT) of the brain/head demonstrated mild underlying volume loss, right posterior infarction, which was not present previously, either sub-acute or old. CT showed no evidence of intracranial hemorrhage, other obvious acute event or vascular calcification. As noted in the exam, the patient did have a slight left-sided visual field loss that she previously had not noticed. However, upon questioning she did note that over the past month when changing lanes to the left while driving she noticed she had to turn her head further to the left to see her blind spot but disregarded this at the time.

**Figure 2:**
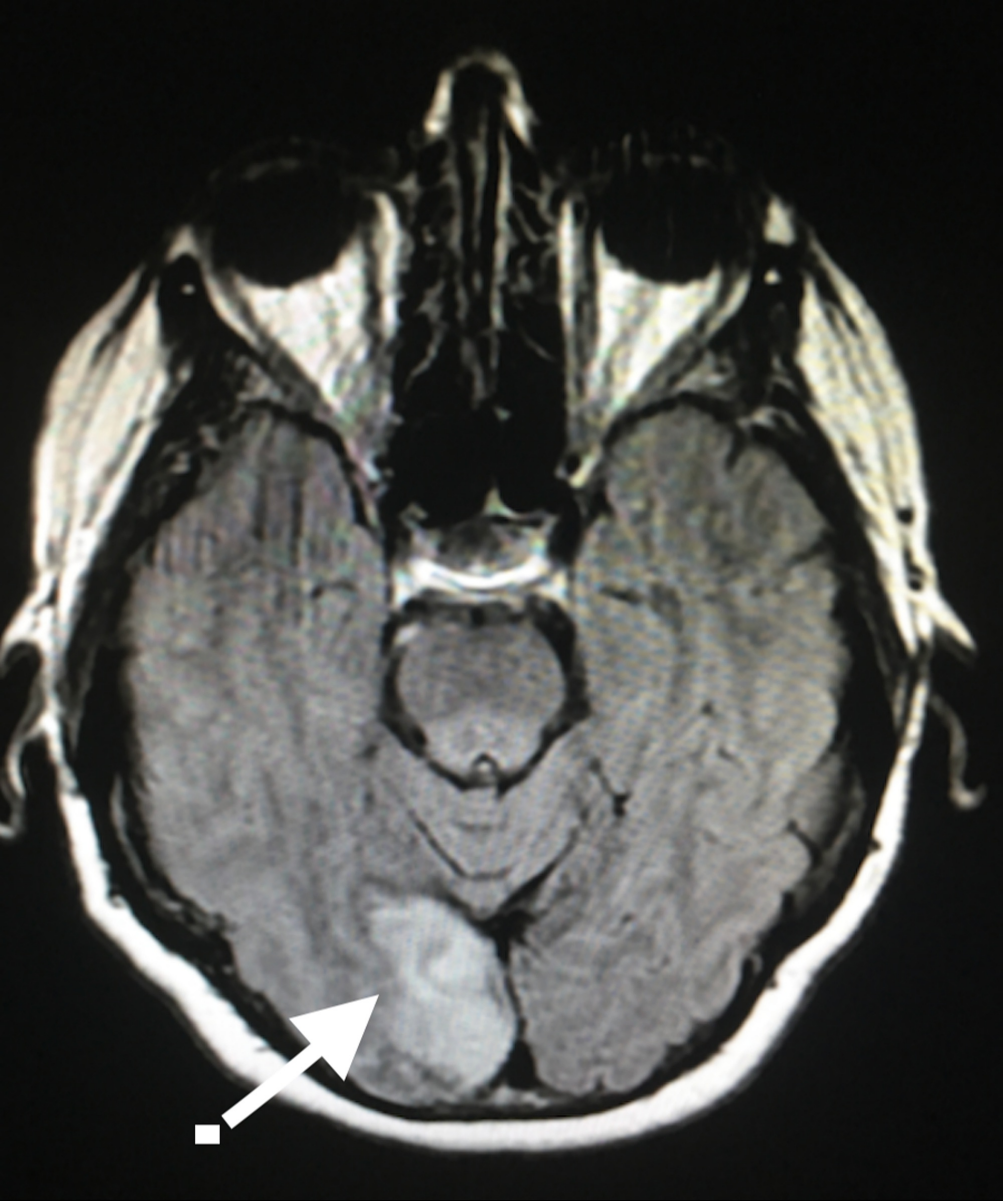
Magnetic Resonance Imaging of Brain Magnetic resonance imaging of brain without contrast demonstrating acute infarct in right medial posterior occipital lobe in the right posterior cerebral artery territory (white arrow).

Our patient's case was discussed with a consulting neurologist at a tertiary center where the patient was ultimately transferred for further evaluation of her new and persistent symptoms. MRI was performed upon admission at the tertiary center (Figure 2) and showed an acute infarct in the right medial posterior occipital lobe in the right posterior cerebral artery territory. The patient's discharge diagnosis was a right occipital infarct and Charles Bonnet syndrome upon discharge from the tertiary center. Unfortunately, patient follow-up and progression of disease is unknown.

## Discussion

Charles Bonnet syndrome is a relatively unknown condition typically associated with a primary ophthalmologic disease, however the disease can less commonly present due to a brain lesion such as a tumor or infarct. A patient presenting with visual hallucinations may initiate a psychiatric work-up. However, if the patient is appropriate and healthy with insight into the unreal nature of their hallucinations and not otherwise impaired with prescription or nonprescription drugs, nonpsychiatric causes should be considered, including Charles Bonnet syndrome [3]. Precisely how and why CBS occurs has not yet been defined. Two theories have been accepted as possible explanations for pathogenesis of the disease. Release theory suggests that a lesion in the visual pathway results in abnormal signals being sent to the visual cortex. The abnormal signals combined with normal signals result in hallucinations. Deprivation theory suggests that a reduction in sensory input leads to the production of spontaneous images from the visual association cortex, resulting in visual hallucinations. This theory is described similarly to the theory of phantom limb pain.

When a stroke occurs in the visual regions of the brain there is an increased risk of visual disturbances, including Charles Bonnet syndrome. In about 20% of strokes, visual or perceptual disturbances may occur. The content of these visual disturbances adds to the mystique of the disorder. Patients diagnosed with CBS have reported seeing basic geometric shapes to vivid structures, distorted faces with headdresses and even finding themselves in different landscapes [1]. It is not understood why or how each patient's hallucinations vary. Unlike most cases of Charles Bonnet syndrome due to primary eye disease, in stroke-induced Charles Bonnet Syndrome the affected persons may retain central vision (visual acuity) even though they may suffer from peripheral visual field loss [3].

Charles Bonnet syndrome has been known for quite some time; however, many medical and health care professionals are unaware of the syndrome. An informal 2010 survey of 343 general practitioners in the Metropolitan area of Sydney, Australia showed that only two admitted to being familiar with Charles Bonnet syndrome [1]. They also expressed having very limited knowledge of the diagnosis and appropriate treatment. There is a high rate of non-reporting of visual hallucinations to care providers due to a fear of being labeled with a psychiatric disorder, not being believed or losing one's independence. Vukicevic and Fitzmaurice found that whereas 21% of Charles Bonnet syndrome patients had not reported their symptoms to anyone, 64% mentioned it to their family members and only 15% had told a health care professional. Directed questioning by the health care professionals regarding hallucinations were essential in identifying patients suffering from Charles Bonnet syndrome [4].

Treatment of Charles Bonnet syndrome is focused on optimizing outpatient eye care. Maximizing improvement of vision may reduce or even cause resolution of symptoms. Since sensory deprivation is a crucial factor in CBS some outpatient goals are to increase sensory stimulus, which may activate the brain in appropriate areas and thus improve symptoms. Stress and anxiety tend to worsen symptoms. No pharmacological agents have been found to be effective. Preliminary studies with electromagnetic stimulation treatments have shown reduction of symptoms temporarily but have not been officially approved for treatment of Charles Bonnet syndrome [1].

## Conclusions

Charles Bonnet syndrome is a clinical condition that is not known to many medical practitioners. On top of being unaware of this condition it may be difficult to obtain a complete history from a patient suffering from Charles Bonnet syndrome due to their fear of being diagnosed with a psychiatric illness. Missing this diagnosis is missing a potential ischemic event or other major neurological condition. Knowledge of Charles Bonnet syndrome as well as the ability to ask directed questions to obtain appropriate history, will be the mainstay of keying a provider into a patient with Charles Bonnet syndrome and is relevant to the practice of emergency medicine.
